# HPat a Decapping Activator Interacting with the miRNA Effector Complex

**DOI:** 10.1371/journal.pone.0071860

**Published:** 2013-08-19

**Authors:** Elisabeth Barišić-Jäger, Izabela Kręcioch, Stefanie Hosiner, Sanja Antic, Silke Dorner

**Affiliations:** Max F. Perutz Laboratories, University of Vienna, Department of Microbiology, Immunbiology and Genetics, Vienna, Austria; Colorado State University, United States of America

## Abstract

Animal miRNAs commonly mediate mRNA degradation and/or translational repression by binding to their target mRNAs. Key factors for miRNA-mediated mRNA degradation are the components of the miRNA effector complex (AGO1 and GW182) and the general mRNA degradation machinery (deadenylation and decapping enzymes). The CCR4-NOT1 complex required for the deadenylation of target mRNAs is directly recruited to the miRNA effector complex. However, it is unclear whether the following decapping step is only a consequence of deadenylation occurring independent of the miRNA effector complex or e.g. decapping activators can get recruited to the miRNA effector complex. In this study we performed split-affinity purifications in *Drosophila* cells and provide evidence for the interaction of the decapping activator HPat with the miRNA effector complex. Furthermore, in knockdown analysis of various mRNA degradation factors we demonstrate the importance of NOT1 for this interaction. This suggests that deadenylation and/or the recruitment of NOT1 protein precedes the association of HPat with the miRNA effector complex. Since HPat couples deadenylation and decapping, the recruitment of HPat to the miRNA effector complex provides a mechanism to commit the mRNA target for degradation.

## Introduction

MicroRNAs (miRNAs) are small non-coding RNAs that commonly regulate gene expression post-transcriptionally by binding to partially complementary sequences in the 3′-UTR of their target mRNAs. Animal miRNAs are key regulators at the translation level but can also accelerate mRNA turnover by recruiting the endogenous mRNA degradation machinery (reviewed in [1], [2]). miRNAs bind to their targets as part of an RNA-protein effector complex, called miRNA-induced silencing complex (miRISC complex). The core of the miRISC complex consists of the miRNA loaded onto an Argonaute protein (AGO) and an Argonaute bound member of the GW182 family (reviewed in [1], [2]). Several proteomic approaches have identified many interactors of the Argonaute [3]–[5] and GW182 [3], [6] proteins, which might modulate the function of the miRISC complex. Recently, the cytoplasmic poly(A)-binding protein PABPC1 [7]–[12] and NOT1, a component of the general CCR4-NOT1 deadenylation complex, have been reported to bind directly to GW182 protein in mammals and *Drosophila* [13]–[15].

The degradation of the majority of animal mRNAs targeted by miRNAs is dependent on the general 5′-to-3′ mRNA degradation machinery [16]–[24]. In this pathway degradation is initiated by deadenylation, followed by decapping and exonucleolytic degradation by XRN1 (reviewed in [25]). In eukaryotes deadenylation involves the consecutive action of two deadenylase complexes. In the first step the PAN2-PAN3 complex shortens the poly(A) tail to about 50–110 nucleotides, while in the second step deadenylation is catalyzed by the CCR4-NOT complex [26]. The CCR4-NOT complex is required for miRNA-mediated mRNA degradation [16], [19]. Even though the PAN2-PAN3 complex binds to the GW182 complex [13], [14] and the overexpression of a catalytically inactive PAN2 mutant slows down deadenylation [18], the PAN2-PAN3 complex is not essential for miRNA-mediated deadenylation [16], [21].

The activity of the decapping enzyme DCP2, which catalyzes the removal of the 5′ -terminal cap (m7G) of mRNAs, requires the binding of decapping activators such as DCP1, HPat (Pat1 in yeast, PatL1 in human), Me31B (Dhh1 in yeast, DDX6/RCK in human), EDC3 or EDC4 (reviewed in [27]). In contrast to deadenylation the role of the decapping step in miRNA-mediated mRNA degradation has been much less investigated. In transcriptome-wide analysis the knockdown of decapping activators resulted in an increase of the levels of predicted or validated miRNA targets [17]. The analysis of the effect of decapping activators on miRNA - mediated degradation is particularly challenging due to the redundancy of decapping activators and the lack of restoration of protein levels upon their depletion [17]. While upon knockdown of decapping activators the levels of deadenylated mRNAs accumulate, these mRNAs are not efficiently translated due to the lack of a poly(A) tail and protein levels are not fully restored [17].

Even though the importance of decapping activators in the miRNA pathway is well established, the mechanism of their recruitment is still an open question. In particular it is unclear whether decapping occurs as a mere consequence of deadenylation or the miRNA effector complex actively recruits decapping activators. In this study we have investigated the co-purification of the miRNA effector components GW182 and AGO1 with the general decapping activator HPat in *Drosophila* S2 cells. In split-affinity purifications using Twin-Strep-tagged AGO1 and TAP-tagged GW182 protein we provide evidence for endogenous HPat to purify in the same complex as GW182 and AGO1. Furthermore, we analyzed the interaction of HPat with GW182 in various knockdown cells. We found the co-purification of HPat to be dependent on AGO1 protein. In addition the interaction of HPat with GW182 is dependent on NOT1 protein suggesting the importance of the NOT1 recruitment to GW182 and/or deadenylation prior to HPat binding. In contrast the knockdown of the decapping activators DCP1 and EDC4 or the exonuclease XRN1 did not affect the interaction of HPat and GW182. These findings suggest the binding of HPat to the miRNA effector complex after the recruitment of NOT1 but before the action of the decapping enzyme.

## Materials and Methods

### Cell Culture, dsRNA, RNA Interference

*Drosophila* S2 cells (Invitrogen) were cultured at 25°C in Schneider’s medium (Lonza) supplemented with 10% heat-inactivated FBS (Sigma), penicillin (100 U/ml Invitrogen), streptomycin (100 µg/ml Invitrogen), 2 mM glutamine (Invitrogen). For the maintenance of stable cell lines 150 µg/ml hygromycin B was added to the media.

RNAi was performed essentially as described in [28]. dsRNAs corresponded to about 700 nt of the coding sequences of the gene of interest. Cells were treated with dsRNA on day 0 and day 4. 30 µg of dsRNA were used per 1–2 Mio. cells per ml serum free media (Express Five SFM, Invitrogen). After 1 hour of soaking 2 ml media supplemented with FBS was added (Express Five SFM supplemented with 10% heat-inactivated FBS, penicillin, streptomycin and glutamine as above). Cells treated with dsRNA against AGO1 were harvested on day 4, while cells treated with dsRNA against NOT1, EDC4, DCP1 or XRN1 were treated twice and harvested on day 7. Cells treated with dsRNA against YFP (yellow fluorescent protein) were used as a control. When treating stable cell lines, the expression of HA-tagged GW182 and Myc-tagged HPat was induced with 0.5 mM CuSO_4_ three days prior to harvesting. The knockdown of the gene of interest was verified by the analysis of the mRNA levels (RT-qPCR). dsRNA was prepared by T7 transcription from PCR templates as described in [28]. Oligonucleotides used to prepare PCR templates for T7 transcription are listed in Table S1 and oligonucleotides for qPCR are listed in Table S2.

### Antibodies and Western Blot Analysis

Polyclonal antibodies against *Drosophila* HPat (NP_650592.1, amino acids 1–490), GW182 (NP_726596.1, amino acids 1–539), and AGO1 (NP_725341.1, amino acids 1–522) were raised in rabbits (Pineda, Berlin) immunized with His-tagged denatured recombinant fusion proteins. For Western blot analysis the polyclonal antibodies were diluted 1∶3,000 and for chemiluminescent detection the primary antibodies were detected with horseradish-peroxidase (HRP) coupled goat anti-rabbit antibody (Jackson Immuno Research, 1∶10,000) and substrates as described in [29].

### Immunoprecipitation Using Anti-HPat or Anti-GW182 Antibodies

*Drosophila* S2 cells were harvested, washed with PBS, and lysed in NET buffer (50 mM Tris/Cl pH 7.5, 150 mM NaCl, 1 mM EDTA and 0.5% NP-40) supplemented with protease inhibitors (Complete, Roche) for 15 min on ice. The clarified lysate was divided and for immunoprecipitation 10 **µ**l purified polyclonal anti-HPat, or anti-GW182, or preimmune sera were added. After one hour overhead rotation at 4°C 40 µl BSA-coated protein A sepharose beads (GE healthcare) were added and the rotation continued for one hour. The beads were washed three times with NET buffer and once with PBS. Proteins were eluted with SDS sample buffer, separated on a SDS-PAGE, and analyzed by Western blotting analysis.

### Immunoprecipitation Using Anti-HA Antibody in Knockdown Cells

After treatment with dsRNA the cells were lysed as above, 20 µl of BSA-coated protein A sepharose beads (GE Healthcare) crosslinked with anti-HA monoclonal antibody (clone 12CA5) were added to lysates and rotated overhead 2 h at 4°C. Beads were washed three times with NET buffer (50 mM Tris pH 7.5, 300 mM NaCl, 1 mM EDTA and 0.5% NP-40) and once with PBS. The proteins were eluted with SDS sample buffer and analyzed by Western blotting with monoclonal anti-HA (clone 12CA5) or monoclonal anti-c-Myc (clone 4A6) antibody. As a secondary antibody Alexa Fluor 680 labeled goat anti-mouse antibody (Invitrogen), IRDye700CW goat anti-mouse antibody (Li-Cor), or IRDye800CW goat anti-mouse antibody (Li-Cor) was used and the membrane scanned using an Odyssey CLx (Li-Cor) instrument. The western blots were quantitated using the Odyssey 2.1 (Li-Cor) or the ImageStudio software (Li-Cor). Linear regression analysis was done using KaleidaGraph. Experiments were performed at least in biological triplicates. For statistical analysis the values were tested for their normal distribution (Shapiro - Wilk) and further analyzed using the Student’s t test.

### Double Pull-down of Twin-Strep-AGO1/Twin-Strep-GFP and TAP-GW182

Stable cell lines expressing TAP-GW182 and Twin-Strep-AGO1 or Twin-Strep-GFP were induced for three days with 0.5 mM CuSO_4_. Cells were lysed in 50 mM Tris/Cl pH 7.5, 150 mM NaCl, 0.5% NP40, protease inhibitor (Complete EDTA free, Roche), 75 µg/ml avidin (IBA), 2 mM Ribonucleoside vanadyl complex (NEB) and 0.2 U/µl RiboLock (Fermentas). Alternatively for RNase A treatment the Ribonucleoside vanadyl complex and RiboLock were substituted by 10 µg/ml RNase A (Fermentas). The clarified lysate was added to MagStrep “type2HC” Strep-Tactin beads (IBA) and rotated for 30 min overhead at 4°C. RNA was isolated from the supernatant after bead binding to check RNA integrity on a denaturing formaldehyde agarose gel. The beads were washed four times with NET buffer and eluted twice in elution buffer (50 mM Tris, 150 mM NaCl, 1 mM EDTA, 10 mM biotin (IBA), pH 8.0). IgG cross-linked Dynabeads were added and rotated overhead for one hour. The beads were washed three times with NET buffer and once with PBS. Proteins were eluted with SDS sample buffer, separated on SDS-PAGE, and analyzed by Western blot analysis. A technical detail, which might be worth mentioning, is our observation that using small size beads (1–4 µm) was crucial for complex purification under RNA maintaining conditions. This observation is consistent with previously published results that large sepharose matrices are inefficient for the isolation of large RNA-protein complexes such as ribosomes [30].

IgG cross-linked beads: 250 µg purified IgG were cross-linked to 10 mg Dynabeads M-270 Epoxy using the Dynabead Antibody Coupling Kit (Invitrogen).

### RNA Isolation and RT-qPCR Analysis

Total RNA was isolated according to manufacturers’ instructions using Tri reagent (Sigma). The RNA was treated Turbo DNAse I (Ambion). cDNA was prepared using random hexamers (Promega) and M-MLV reverse transcriptase (Moloney murine leukemia virus reverse transcriptase, Promega) according to the manufacturers’ protocol. qPCR analysis was performed on a StepOne real time PCR system (Applied Biosystems) using gene-specific primer pairs (listed in Table S2) and Power SYBR green PCR master mix (Applied Biosystems). The qPCR results were analyzed by the comparative threshold cycle method [31] using *rp49* as an internal control gene. For statistical analysis the values were tested for their normality (Shapiro-Wilk) and a Student’s t test was performed to analyze their significance.

## Results

### The Co-purification of HPat and GW182

**Figure 1:**
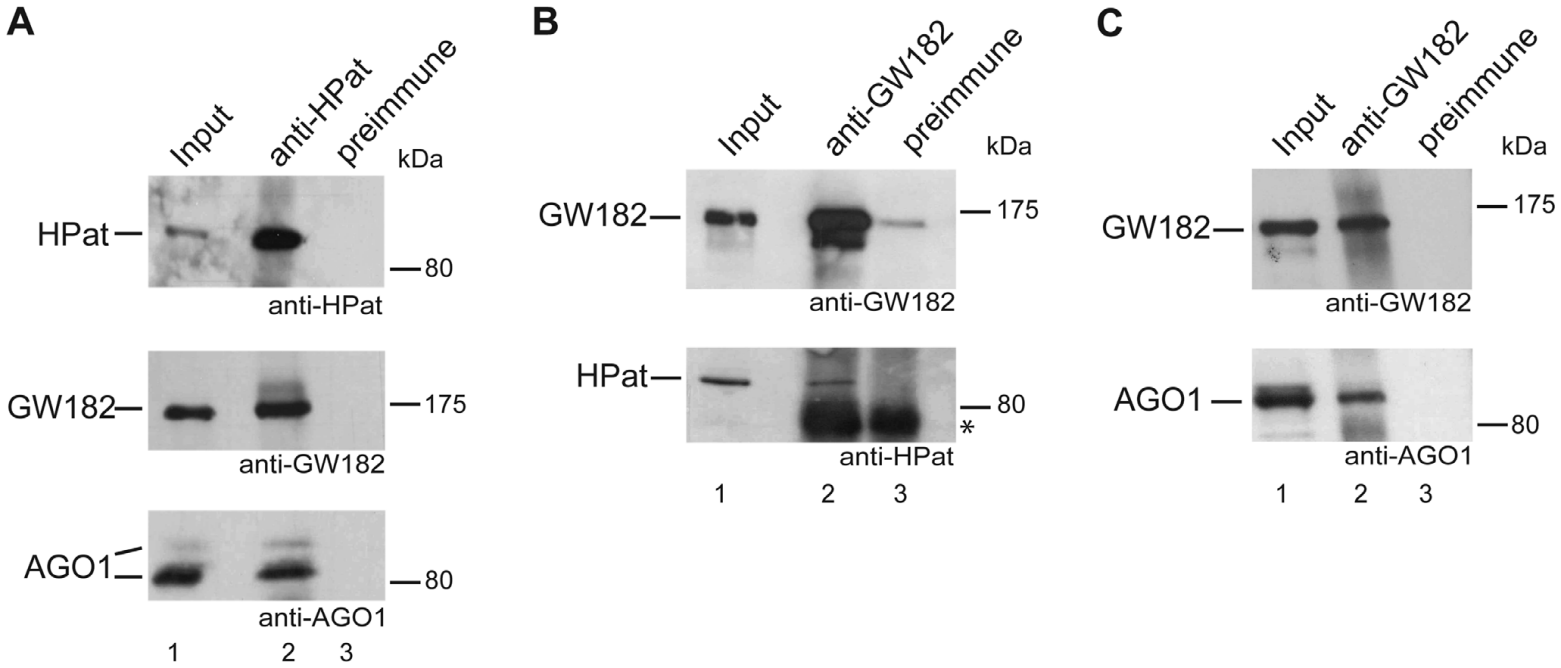
The interaction of HPat and GW182 protein. Immunoprecipitation analysis of *Drosophila* S2 cell lysates using anti-HPat (**A**) or anti-GW182 (**B, C**) antibodies or preimmune sera. Input (lane 1) and immunoprecipitates (lanes 2, 3) were separated on SDS-PAGE and analyzed by Western blot analysis using anti-HPat, anti-GW182 or anti-AGO1 antibody. In A) and B) 1.5% of the input (total clarified cell lysate) and 40% of the immunoprecipitate were separated on a SDS-PAGE, while in C) 2.5% of the input and only 10% of the immunoprecipitate were separated. The asterix indicates cross-reactivity of the secondary antibody with the immunoglobulin heavy chain of the antibody used for immunoprecipitation.

Recently, we provided evidence for the co-immunopurification of the epitope-tagged decapping activator HPat with the miRNA effector component HA-GW182 in *Drosophila* cells [32]. In this study, we further expand the former analysis using antibodies against endogenous HPat and GW182 in immunoprecipitation experiments. Specifically, anti-HPat antibody was added to *Drosophila* S2 cell lysates and the immunoprecipitate analyzed by western blot analysis using anti-HPat, anti-GW182 or anti-AGO1 antibody. Both, GW182 and AGO1 proteins co-purified in immunoprecipitations using anti-HPat antibody but not with preimmune sera (Figure 1A, lane 2 and 3). In addition, we tested whether HPat protein would also co-purify in immunoprecipitates of anti-GW182 antibody from *Drosophila* S2 cell lysates. The immunoprecipitates using anti-GW182 antibody or preimmune sera were analyzed by western blot analysis (Figure 1B and 1C). Again HPat purified specifically in complexes with GW182 but not with preimmune sera (Figure 1B, lane 2 and 3) confirming our results using epitope-tagged GW182 protein [32]. As a control we also tested for the co-purification of AGO1 with GW182 protein (Figure 1C, lane 2). Interestingly, in this series of immunoprecipitations HPat not only co-purified GW182 but also AGO1 protein suggesting the interaction of HPat with GW182 and AGO1 in the same or in different complexes.

### The Interaction of HPat with the miRNA Effector Complex

**Figure 2:**
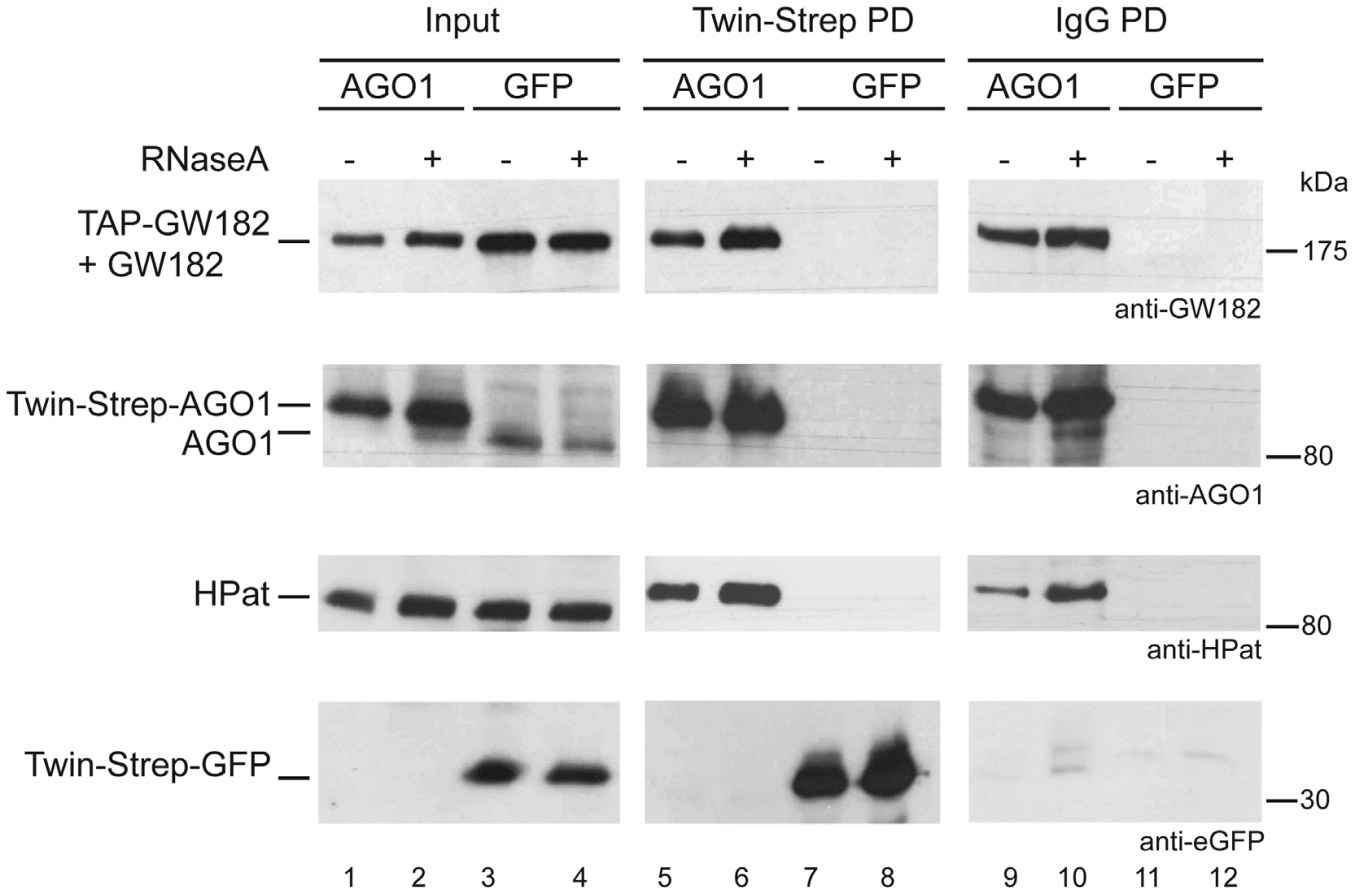
Split-affinity purification of Twin-Strep-AGO1/GFP and TAP-GW182. Cells co-expressing TAP-GW182 and Twin-Strep-AGO1 or Twin-Strep-GFP were lysed under RNA maintaining (- RNase lanes 1, 3, 5, 7, 9, and 11) or RNase A (+ lanes 2, 4, 6, 8, 10, and 12) conditions. As input 0.5% of the total cell lysate was loaded (lanes 1 to 4). In the first pulldown the lysate was incubated with Strep-Tactin beads isolating Twin-Strep-AGO1 or Twin-Strep-GFP complexes. 10% of the biotin eluates was analyzed by western blot analysis in lanes 5 to 8 using anti-GW182, anti-AGO1, anti-HPat or anti-GFP antibodies. The remaining biotin eluate was further purified in the second pulldown using IgG-coupled beads and proteins analyzed (50% of the pulldown) in lanes 9 to 12 by western blot analysis.

In the next step we tested the possibility of HPat to associate with AGO1 and GW182 protein in the same complex. We performed split-affinity purifications using two differently epitope tagged proteins in two consecutive purification steps. We co-expressed Twin-Strep tagged AGO1 and TAP-tagged GW182 in a stable *Drosophila* S2 cell line. In the first purification step we isolated Twin-Strep-tagged AGO1 complexes from cell lysates using Strep-Tactin beads. As a control we used a stable cell line co-expressing Twin-Strep tagged GFP and TAP-tagged GW182 for complex purification. Input samples (Figure 2, lane 1 and lane 3) and biotin eluates (Figure 2, lane 5 and lane 7) were analyzed by western blot analysis using anti-GW182, anti-AGO1, anti-HPat or anti-GFP antibody. As expected the Twin-Strep-tagged AGO1 but not endogenous AGO1 protein was detected in the first pull-down (Figure 2, lane 5). In addition also GW182 and HPat co-purified in the Twin-Strep-AGO1 complexes. The TAP-GW182 protein and the GW182 protein are detected in a single band by western blot analysis. In the Supporting Figure S1A the input samples are separated on SDS-PAGE resolving the epitope-tagged and endogenous GW182 (lane 1). In the eluates of a pulldown with IgG-coupled beads, which bind to the protein A moiety of the TAP-tag, only the isolated TAP-GW182 but not endogenous GW182 was detected (Supporting Figure S1A, lane 2). In the control pull-down of Twin-Strep-tagged GFP with Strep-Tactin beads none of the factors (GW182, AGO1 or HPat) co-purified in the biotin eluate. Overall, the analysis of the first eluate confirmed the co-purification of HPat with GW182 and AGO1 in pull-downs using tagged-AGO1 protein.

Since we co-expressed two differently tagged proteins we added a second purification step and tested whether this potential mix of various different complexes also includes a complex with all three factors GW182, AGO1 and HPat. We used the isolated Twin-Strep-tagged AGO1 complexes (Figure 2, lane 5) in a second pull-down with IgG coupled beads binding to TAP-GW182. Thus only complexes with both Twin-Strep-tagged AGO1 and TAP-tagged GW182 are isolated in the second step. In order to control for unspecific enrichment we used the Twin-Strep-tagged GFP eluate (Figure 2, lane 7). Figure 2 lane 9 shows the co-purification of HPat also in the second purification step isolating TAP-GW182 complexes. In the control eluate of Twin-Strep-GFP complexes none of the proteins GW182, AGO1, HPat or Twin-Strep-GFP was detected on IgG beads (Figure 2, lane 11). Thus at least a subpopulation of the isolated AGO1-GW182 complexes has interacted with HPat.

HPat is a general decapping activator binding to mRNAs when promoting their degradation. Therefore, the association of HPat with AGO1 and GW182 could be a protein - protein interaction or might be mediated by RNA interactions. Thus as a control we performed the same split-affinity purification as described above from cell lysates treated with RNase A prior to purification (Figure 2, lanes 2, 4, 6, 8, 10 and 12). To check for RNA integrity and successful RNase digestion the RNA of the supernatant after binding to Strep-Tactin beads was isolated and analyzed on a denaturing formaldehyde gel (Supporting Figure S1B). Again endogenous HPat protein co-purified with both the Twin-Strep-AGO1 complex (Figure 2 lanes 6) and the TAP-GW182 complexes (Figure 2 lane 10). Thus the interaction of HPat with GW182 and AGO1 is not sensitive to RNase treatment and therefore not mediated by long-range RNA interactions. This result is also consistent with our previous observation that the co-purification of epitope-tagged HPat with HA-GW182 is not sensitive to RNase treatment [32].

Overall in this series of split-affinity purifications we could demonstrate the co-purification of HPat with both AGO1 and GW182 in the same complex. This result strongly suggests the recruitment of the decapping activator HPat to the miRNA effector complex.

### The Interaction of HPat with GW182 is Dependent on AGO1

**Figure 3:**
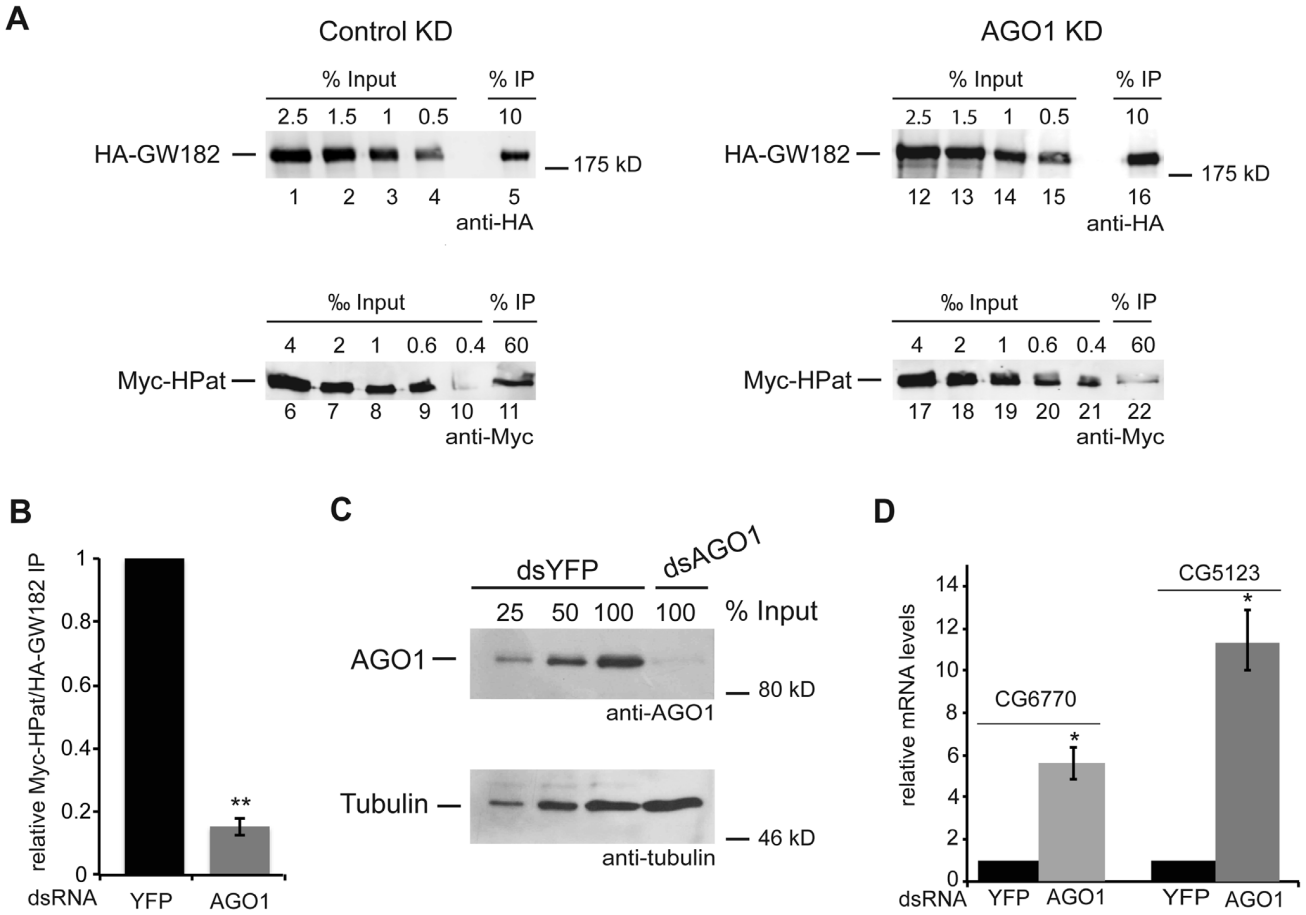
Co-purification of HPat with GW182 in AGO1 knockdown cells. **A:** Cells stably expressing HA-GW182 and Myc-HPat were treated for four days with dsRNA against YFP (control KD) or AGO1 (AGO1 KD). Protein complexes were immunoprecipiated from cell lysates using anti-HA antibody. Increasing amounts of the input sample and immunoprecipitates (IP) were analyzed by western blot analysis using anti-HA (Inputs lanes 1–4, 12–15, and IPs lanes 5 and 16) or anti-c-myc antibody (Inputs lanes 6–10, 17–21 and IPs lanes 11 and 22). The percentage of total cell lysate loaded in input lanes or the percentage of the total IP are indicated. **B:** Quantitative analysis of the western blot in (A). The amount of Myc-HPat/HA-GW182 in the IP was normalized and the value for the control IP set to 1. **C:** Knockdown efficiency of AGO1. Cell lysate of AGO1 knockdown cells and various amounts of control cell lysate were analyzed by western blot analysis. Tubulin was used as a loading control. **D:** Upregulation of endogenous miRNA targets *CG5123* and *CG6770* in AGO1 knockdown cells. Total RNA from input samples of (A) were analyzed by RT-qPCR and normalized to *rp49* levels. The values of dsYFP treated cells were set to 1. In all figures bars represent mean values and error bars standard deviations of at least three biological replicates. Statistical analysis was performed using the Student’s *t* test and significance values are as follows: *, p<0.002; **, p<0.0001.

As a next step we tested the importance of AGO1 for the co-purification of HPat with GW182. Since the polyclonal antibodies were not suitable for quantitative western blot analysis we used our well-characterized system with epitope tagged Myc-HPat and HA-GW182 [32] and tested for the co-purification of Myc-HPat with HA-GW182 in AGO1 knockdown cells. Both proteins, Myc-HPat and HA-GW182 were co-expressed in stable *Drosophila* S2 cells. Prior to the immunopurification of complexes the cells were treated with dsRNA against AGO1 or yellow fluorescent protein (YFP) as a control for unspecific effects due to the dsRNA treatment. HA-GW182 complexes were immunoprecipitated from cell lysates using anti-HA antibody. Increasing amounts of input samples and immunoprecipitates were analyzed by western blot analysis using anti-HA or anti-Myc antibody (Figure 3A). The western blots were quantitated (Supporting Figure S2), the ratio of Myc-HPat/HA-GW182 in the immunoprecipitates was calculated and the Myc-HPat/HA-GW182 ratio of AGO1 treated cells normalized to YFP control cells (Figure 3B). In AGO1 knockdown cells the Myc-HPat/HA-GW182 ratio was decreased by 84.6±2.8% (Figure 3B). This significant decrease of the amount of HPat co-purifying with GW182 in AGO1 knockdown cells underlines the importance of AGO1. The information of the quantitative western blot analysis was used to calculate the expression of Myc-HPat/HA-GW182 in input samples of YFP and AGO1 knockdown cells. Supporting Figure S3A shows similar input ratios of Myc-HPat/HA-GW182 in both lysates. We assessed the knockdown of AGO1 by western blot analysis (Figure 3C). In addition we analyzed the levels of two endogenous mRNAs, *CG6770* and *CG5123*, by RT-qPCR in dsYFP and dsAGO1 treated cells (Figure 3D). Consistent with previous reports [16], [19], the mRNA levels of *CG6770* and *CG5123* were increased in our AGO1 knockdown cells. Thus we used these two mRNAs to monitor the effect of the knockdown on the endogenous miRNA pathway.

### The Interaction of HPat with GW182 is Dependent on NOT1 but not on EDC4/DCP1 or XRN1

**Figure 4:**
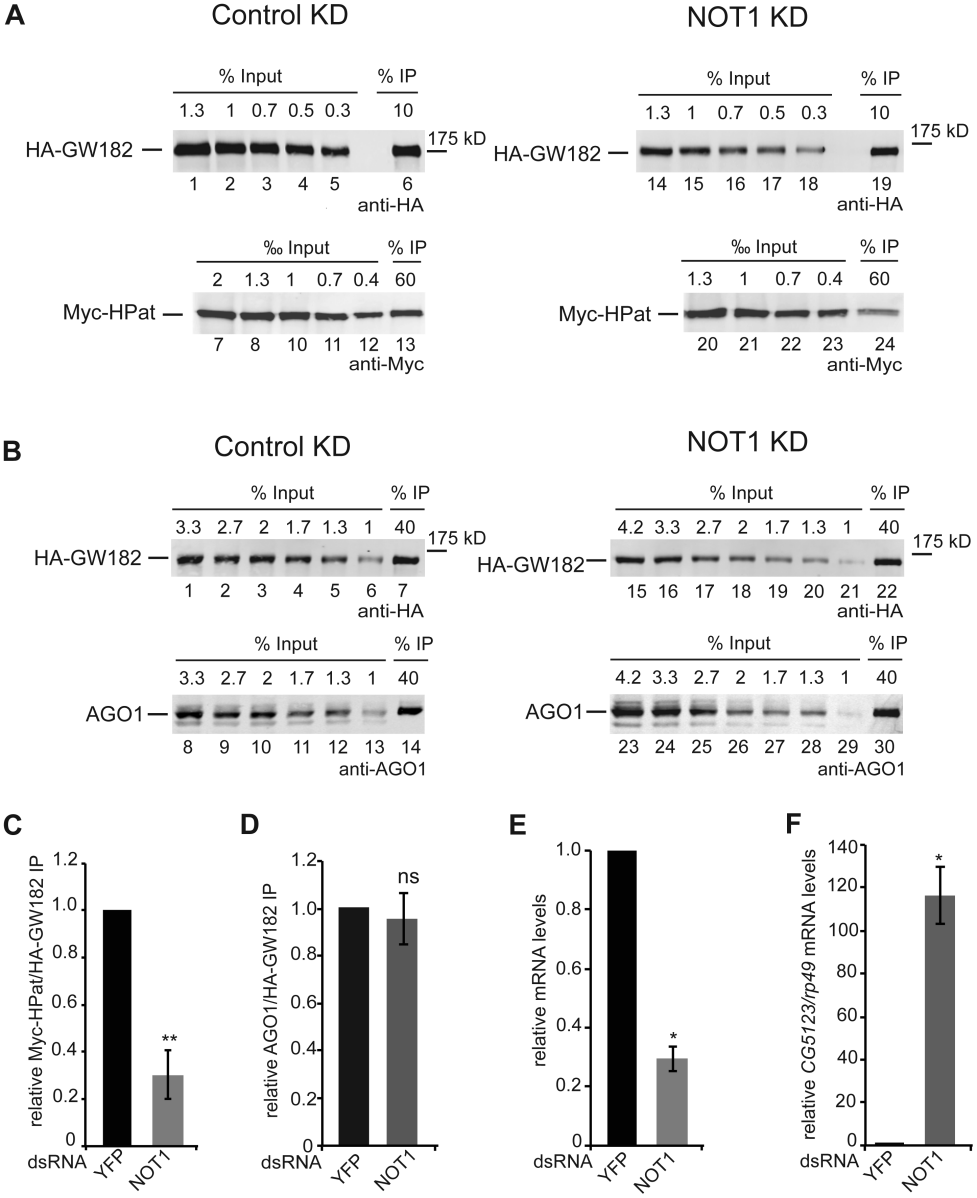
Co-purification of HPat (A) or AGO1 (B) with GW182 in NOT1 knockdown cells. **A, B:** Protein complexes were immunoprecipiated using monoclonal anti-HA antibody from cell lysates. Cells stable expressing HA-GW182 and Myc-HPat were treated with dsRNA against YFP (control KD) or NOT1 (NOT1 KD). Increasing amounts of the input sample and immunoprecipitates (IP) were analyzed by western blot analysis using anti-HA (Input in A: lanes 1–5 and 14–18, in B: lanes 1–6 and 15–21. IPs in A lanes 6 and 19, in B lanes 7 and 22), anti-c-myc (Input in A: lanes 7–12 and 20–23. IPs in A lanes 13 and 24) or anti-AGO1 antibody (Input in B: lanes 8–13 and 23–29. IPs in B lanes 14 and 30). The percentage of total cell lysate loaded in input lanes or the percentage of the total IP are indicated. **C, D:** The amount of Myc-HPat/HA-GW182 (**C**) or AGO1/HA-GW182 (**D**) in immunoprecipitates (IP) from lysates of control and NOT1 knockdown cells. The IP was normalized (Supporting Figure S4) and the value of the control IP set to 1. **E:** Analysis of *NOT1* mRNA levels in knockdown cells compared to control cells treated with dsYFP RNA. The levels of *NOT1* mRNA in total RNA of input samples were analyzed by RT-qPCR and normalized to *rp49* mRNA levels. The values of dsYFP treated cells were set to 1. **F:** Upregulation of endogenous miRNA targets in knockdown cells. Total RNA of input samples were analyzed by RT-qPCR for changes of *CG5123* mRNA levels in NOT1 knockdown cells. mRNA levels were normalized to *rp49* mRNA levels. The values of dsYFP treated cells were set to 1. Statistical analysis was performed using the Student’s *t* test and significance values are as follows: ns, not significant; *, p<0.005; **, p<0.001.

It is well established that GW182 directly interacts with NOT1 of the deadenylation complex CCR4-NOT in *Drosophila* and mammalian cells [13]–[15]. In addition in *Drosophila* cells the decapping activator HPat was shown to interact with the CCR4-NOT complex [8]. Thus we further analyzed the HPat interaction with GW182 in NOT1 knockdown cells. Similar as for the AGO1 knockdown cells, *Drosophila* S2 cells stable expressing Myc-HPat and HA-GW182 were treated with dsRNA against NOT1 or dsRNA against YFP as a control. Cell lysates were incubated with anti-HA antibody and the immunoprecipitates analyzed by quantitative western blot analysis using anti-HA or anti-Myc antibody (Figure 4A). The calculated ratio of Myc-HPat/HA-GW182 in the immunoprecipitates (Supporting Figure S4A) was 69.7±10.2% decreased in NOT1 knockdown cells compared to control cells (Figure 4C). However, the Myc-HPat/HA-GW182 ratio in input samples was about three times higher in NOT1 knockdown cells compared to control cells (Supporting Figure S3A). Analysis of the HA-GW182 and Myc-HPat protein levels using Tubulin as a loading control showed a decrease of HA-GW182 in NOT1 knockdown cells (Supporting Figure S3B). That some epitope-tagged proteins can be less well expressed in NOT1 knockdown cells was previously reported [33]. It was important to test whether the decrease in the co-purification of HPat in NOT1 knockdown cells could be due to the reduced expression levels of HA-GW182. Since the levels of AGO1 protein showed the same tendency in NOT1 knockdown cells as Myc-HPat (Supporting Figure S3B, S3C), we tested the co-purification of AGO1 with HA-GW182. Thus the immunoprecipitates of anti-HA antibody in NOT1 knockdown cells and control cells were analyzed by quantitative western blot analysis using anti-HA or anti-AGO1 antibody (Figure 4B, Supporting Figure S4B). The calculated ratio of AGO1/HA-GW182 in the immunoprecipitates (Figure 4D) was not affected. Thus we concluded that the decrease of Myc-HPat co-purifying with HA-GW182 is a consequence of the lack of NOT1 protein and not an indirect effect of the decreased HA-GW182 expression level. In order to assess the knockdown efficiency we first analyzed the mRNA levels of *NOT1* mRNA relative to *rp49* mRNA by RT-qPCR of total RNA from input samples (Figure 4E). Secondly, we analyzed the levels of *CG5123* mRNA by RT-qPCR. *CG5123* mRNA was shown to be upregulated in AGO1 and NOT1 knockdown cells [19]. Figure 4F shows a significant increase of *CG5123* mRNA in NOT1 knockdown cells.

**Figure 5:**
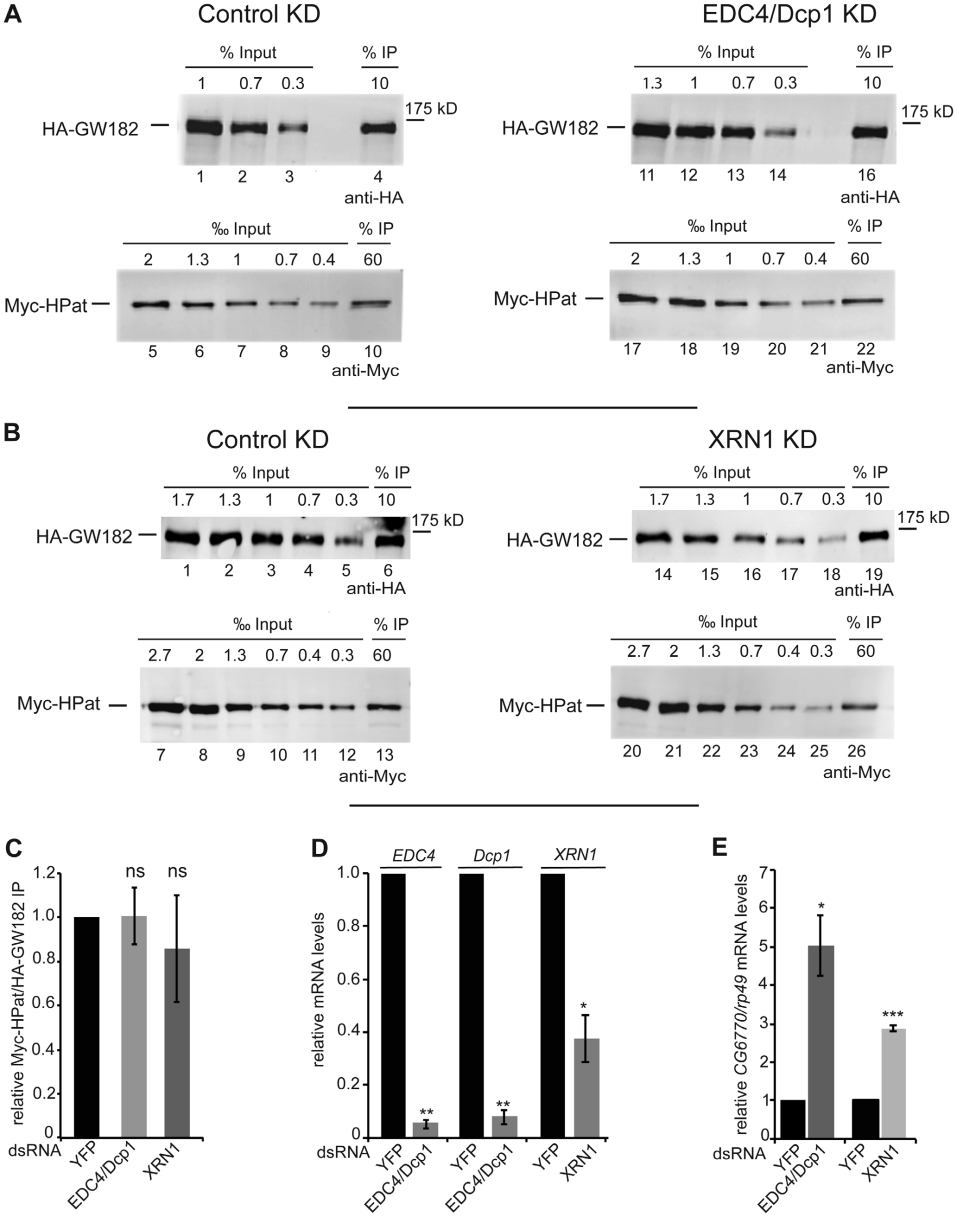
Co-purification of HPat with GW182 in EDC4 and Dcp1 (A), or XRN1 (B) knockdown cells. **A, B:** Protein complexes were immunoprecipiated using monoclonal anti-HA antibody from cell lysates. Cells stable expressing HA-GW182 and Myc-HPat were treated with dsRNA against YFP (control KD), EDC4 and Dcp1 (EDC4/Dcp1 KD, **A**) or XRN1 (XRN1 KD, **B**). Increasing amounts of the input sample and immunoprecipitates (IP) were analyzed by western blot analysis using anti-HA (Input in A: lanes 1–3 and 11–14, in B: lanes 1–5 and 14–18. IPs in A: lanes 4 and 16, in B: lanes 6 and 19) or anti-c-myc antibody (Input in A: lanes 5–9 and 17–21, in B: lanes 7–12 and 20–25. IPs in A: lanes 10 and 22, in B: lanes 13 and 26). The percentage of total cell lysate loaded in input lanes or the percentage of the total IP are indicated. **C:** The amount of Myc-HPat/HA-GW182 in immunoprecipitates (IP) from lysates of control, EDC4 and Dcp1, or XRN1 knockdown cells. The IP was normalized (Supporting Figure S5) and the value of the control IP set to 1. **D:** Analysis of *EDC4, Dcp1,* and *XRN1* mRNA levels. The levels of *EDC4, Dcp1,* and *XRN1* mRNA in total RNA of input samples were analyzed by RT-qPCR and normalized to *rp49* mRNA levels. The values of dsYFP treated cells were set to 1. **E:** Upregulation of endogenous miRNA targets in knockdown cells. *CG6770* mRNA levels in total RNA of EDC4/Dcp1, XRN1, and YFP knockdown cells were analyzed by RT-qPCR. mRNA levels were normalized to *rp49* mRNA levels. The values of dsYFP treated cells were set to 1. Statistical analysis was performed using the Student’s *t* test and significance values are as follows: ns, not significant; *, p<0.02; **, p<0.001; ***, p<0.0001.

While the knockdown of NOT1 in *Drosophila* cells affects the deadenylation of bulk mRNAs [34], [35], the simultaneous knockdown of at least two decapping activators such as EDC4 and DCP1 prevents decapping but allows for the deadenylation of mRNAs [17], [36]. Additionally, in knockdown cells of the exonuclease XRN1 mRNA degradation is inhibited mainly as a consequence of the inhibition of decapping [37]. In order to further analyze the effect of factors acting downstream of deadenylation during mRNA degradation we monitored the interaction of GW182 and HPat in double-knockdown cells of EDC4 and DCP1 or knockdown cells of XRN1. In anti-HA immunoprecipitates of EDC4/DCP1 or XRN1 treated cells the ratios of Myc-HPat/HA-GW182 were not significantly changed compared to dsYFP treated control cells (Figure 5 A–C, Supporting Figure S5). The ratio of Myc-HPat/HA-GW182 in input samples was unchanged in these knockdowns (Supporting Figure S3A). Again the knockdown of *EDC4, DCP1* and *XRN1* mRNA was assessed by RT-qPCR (Figure 5D) and the abrogation of the miRNA target *CG6770* was monitored (Figure 5E).

In summary we detect a significant decrease in the co-immunopurification of HPat with GW182 in NOT1 knockdown cells but not in EDC4/DCP1 or XRN1 knockdown cells. This indicates the importance of deadenylation and/or NOT1 binding for the recruitment of HPat to the miRNA effector complex. Furthermore, it places the interaction of HPat and GW182 before decapping since knockdown of additional decapping activators does not affect the interaction.

## Discussion

mRNA degradation mediated by miRNAs is dependent on the general mRNA degradation machinery required for the cytoplasmic 5′ - to - 3′ degradation of bulk mRNAs. Thus these mRNAs targeted by miRNAs are deadenylated followed by decapping and exonucleolytic degradation by XRN1 [16]–[22]. Recently, it was established that GW182, a key component of the miRNA effector complex, directly binds NOT1 of the deadenylase complex CCR4-NOT1 in *Drosophila* and mammalian cells [13]–[15]. However, it is unknown whether the following decapping step is only a consequence of deadenylation and occurs independent of the miRNA effector complex. In this study we show that HPat, a general decapping activator, interacts with the miRNA effector complex. Furthermore, this interaction is not only dependent on AGO1 but also on the NOT1 protein. These findings suggest a recruitment of the decapping activator HPat to the miRNA effector complex after NOT1 binding. Thus strongly supporting the idea of GW182 as a binding platform for modulating the miRNA response [1]. Both human Pat1b and *Drosophila* HPat are known to couple deadenylation and decapping [36], [38]. Thus the recruitment of HPat to the miRNA effector complex will promote decapping and commit the deadenylated mRNA target for further degradation.

In this study we investigated the co-purification of the general decapping activator HPat with the miRNA effector components GW182 and AGO1. In immunoprecipitation experiments using endogenous anti-HPat antibody we could demonstrate the co-purification of both endogenous GW182 and AGO1 protein. Furthermore, in split-affinity purifications endogenous HPat co-purified with AGO1 and GW182 after two consecutive complex purifications utilizing Twin-AGO1 followed by TAP-GW182 protein. Thus HPat, GW182 and AGO1 are in one complex at some point during miRNA-mediated mRNA degradation. Additionally, in knockdown analysis the interaction of HPat with GW182 is strongly dependent on AGO1 and NOT1 protein but not on additional decapping activators or the exonuclease XRN1. Unfortunately, due to insolubility of the *Drosophila* HPat protein and its fragments we could not test whether the interaction of HPat with the miRNA effector complex is mediated by a direct binding to AGO1, GW182 or NOT1 protein (data not shown). However, artificial tethering of GW182 in AGO1 knockdown cells induces mRNA degradation and therefore bypasses the requirement for AGO1 [16]. Thus indicating that AGO1 is not likely to play a crucial role in the recruitment of HPat to the miRNA effector complex. The strong reduction of the co-purification of HPat with GW182 in NOT1 but not in EDC4/DCP1 or XRN1 knockdown cells suggests that binding of HPat to the miRNA effector complex occurs after NOT1 binding and/or deadenylation but before mRNA decapping. In contrast to the degradation of bulk mRNAs a recruitment of HPat to the miRNA effector complex by NOT1 would have to accommodate GW182 and AGO1. Interestingly, it was previously observed that miRNA-mediated mRNA degradation, which is dependent on GW182, might be slightly different than the degradation of bulk mRNAs [36]. While generally HPat interacts with Me31B through a conserved N-terminal sequence, this domain is dispensable for GW182 - dependent mRNA degradation [36]. This observation is also consistent with our previous results were Me31B did not co-purify with GW182 in *Drosophila* cells [32]. Future experiments will certainly have to address the timing of HPat recruitment and its direct interaction partners in the miRNA effector complex to yield a more detailed mechanistic model.

HPat is conserved in eukaryotes and it is a general decapping activator as its orthologues in yeast (Pat1, [39]–[41]) and human cells (PatL1 or Pat1b [38], [42]). In *Drosophila* cells HPat interacts with additional decapping factors such as Me31B, Lsm1-7 complex, and the decapping enzyme DCP2 but also the deadenylase complex CCR4-NOT [36]. Thus HPat was suggested to have an important role in coupling the deadenylation and the decapping of mRNAs in the general 5′-to-3′ mRNA degradation pathway [36]. In addition HPat interacts genetically with CCR4 and AGO1 but not Dcp2 to control synaptic terminal growth in *Drosophila* [43]. That artificial tethering of HPat to an mRNA is sufficient for destabilization further indicates the importance of HPat in the process of mRNA degradation [36]. Similar results have also been obtained for the human Pat1b [38]. Furthermore, in yeast Pat1 is a key regulator promoting the transition of mRNAs from their translational active form to the state of mRNA degradation [44]–[46]. In particular, Pat1 also has been characterized to directly repress translation during translation initiation upstream of 48S formation [46]. Thus it is tempting to speculate that the recruitment of HPat to the miRNA effector complex could provide a mechanism to ensure the transition from mRNA translation to degradation. Furthermore, a selective recruitment of HPat to the miRNA effector complex could specifically commit some but not all mRNAs targeted by miRNAs for degradation.

## Supporting Information

Figure S1 **Binding of TAP-GW182 to IgG beads (A) and RNA integrity check of pulldowns (B). A:** Expression of TAP-GW182 and binding to IgG-coupled beads. In lane 1 0.5% input (total cell lysate from Figure 2, lane 1) and in lane 2 25% of the eluate from IgG coupled beads (Figure 2, lane 9) were separated on SDS-PAGE and analyzed by western blot analysis using anti-GW182 antibody. **B:** Total RNA was isolated from supernatants after binding of the lysate to Strep-Tactin beads in experiment Figure 2. The RNA was analyzed on a denaturing formaldehyde agarose gel. Specifically, the RNAs in lane 1, 2, 3, and 4 were isolated from supernatants of Figure 2, lane 5, 6,7, and 8 respectively. In Drosophila 28S rRNA is hydrolysed upon heat denaturation into two fragments, which migrate similar to 18S rRNA (Greenberg, J.R. (1969) Synthesis and properties of ribosomal RNA in Drosophila. J Mol Biol, 46, 85–98.).
(TIF)

Figure S2 **Quantitative analysis of the western blots shown in** **Figure 3A** **.** Graphs for control cells treated with dsYFP RNA are shown in (A) and AGO1 knockdown cells in (B). The signal intensities were obtained using the Odyssey 2.1 (Li-Cor) and plotted against the amount of cell lysate. The amount of Myc-HPat or HA-GW182 in the immunoprecipitate was calculated relative to the amount of cell lysate in the input sample.
(TIF)

Figure S3 **A, C:** Relative expression levels of Myc-HPat/HA-GW182 (A) or AGO1/HA-GW182 (C) in different knockdown cells. The linear regression line of the quantitative input analysis of all biological replicates including Supporting Figures S2, S4 and S5 were used to calculate the ratio of Myc-HPat (A) or AGO1 (C) to HA-GW182 in input samples. As in all manuscript figures the bars represent the mean values of at least three independent biological replicates and the error bars the standard deviations. **B:** Protein levels of Myc-HPat, HA-GW182, and Tubulin in NOT1 and YFP knockdown cells. Increasing amounts of cell lysates from control cells (lanes 1–7) and NOT1 knockdown cells (lanes 8–13) were analyzed by western blot analysis using anti-HA, anti-myc, anti-AGO1 or anti-Tubulin antibody. The percentage of total cell lysate loaded is indicated. The graph below shows the quantitative analysis of the western blot. The signal intensities were obtained using the Odyssey 2.1 or ImageStudio (Li-Cor) and plotted relative to the amount of cell lysate. Values obtained from the linear regression were used to normalize HA-GW182, Myc-HPat, or AGO1 to Tubulin. These normalized values were used to calculate the ratio of HA-GW182, Myc-HPat, or AGO1 in NOT1 knockdown cells to control cells. For statistical analyses in A–C the Student’s t test was performed and the significances are as follows: ns, not significant; *, p<0.05; **, p<0.01.
(TIF)

Figure S4 **Quantitative analysis of the western blots shown in** **Figure 4A** **(A) and** **Figure 4B** **(B).** The signal intensities were obtained using the Odyssey 2.1 (Li-Cor) and plotted relative to the amount of cell lysate. The amount of Myc-HPat or HA-GW182 in the immunoprecipitate was calculated relative to the amount of cell lysate in the input sample.
(TIF)

Figure S5 **Quantitative analysis of the western blots shown in** **Figure 5A** **(A) and** **Figure 5B** **(B).** The signal intensities were obtained using the Odyssey 2.1 (Li-Cor) and plotted relative to the amount of cell lysate. The amount of Myc-HPat or HA-GW182 in the immunoprecipitate was calculated relative to the amount of cell lysate in the input sample.
(TIF)

Table S1 **Primer sequences for PCR fragments for dsRNA synthesis.**
(PDF)

Table S2 **Primer sequences for qPCR analysis.**
(PDF)
