# Supplementary figures and images for: HPat a Decapping Activator Interacting with the miRNA Effector Complex
Source: PLoS One. 2013 Aug 19;8(8):e71860. doi: 10.1371/journal.pone.0071860 (PMC3747071; doi:10.1371/journal.pone.0071860)

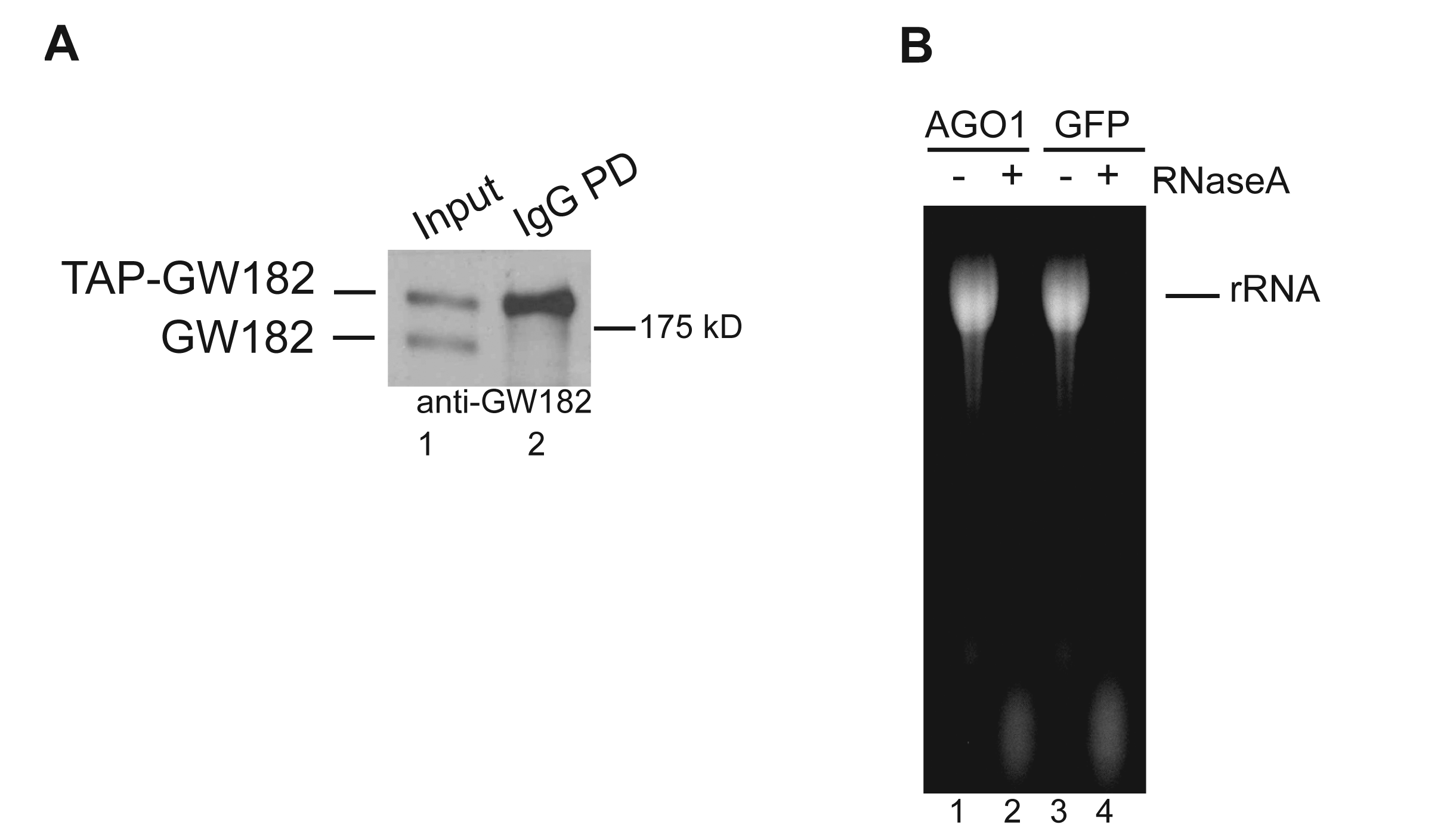

Supplement: Figure S1 — Binding of TAP-GW182 to IgG beads (A) and RNA integrity check of pulldowns (B). A: Expression of TAP-GW182 and binding to IgG-coupled beads. In lane 1 0.5% input (total cell lysate from Figure 2, lane 1) and in lane 2 25% of the eluate from IgG coupled beads (Figure 2, lane 9) were separated on SDS-PAGE and analyzed by western blot analysis using anti-GW182 antibody. B: Total RNA was isolated from supernatants after binding of the lysate to Strep-Tactin beads in experiment Figure 2. The RNA was analyzed on a denaturing formaldehyde agarose gel. Specifically, the RNAs in lane 1, 2, 3, and 4 were isolated from supernatants of Figure 2, lane 5, 6,7, and 8 respectively. In Drosophila 28S rRNA is hydrolysed upon heat denaturation into two fragments, which migrate similar to 18S rRNA (Greenberg, J.R. (1969) Synthesis and properties of ribosomal RNA in Drosophila. J Mol Biol, 46, 85–98.). (TIF) [file pone.0071860.s001.tif]

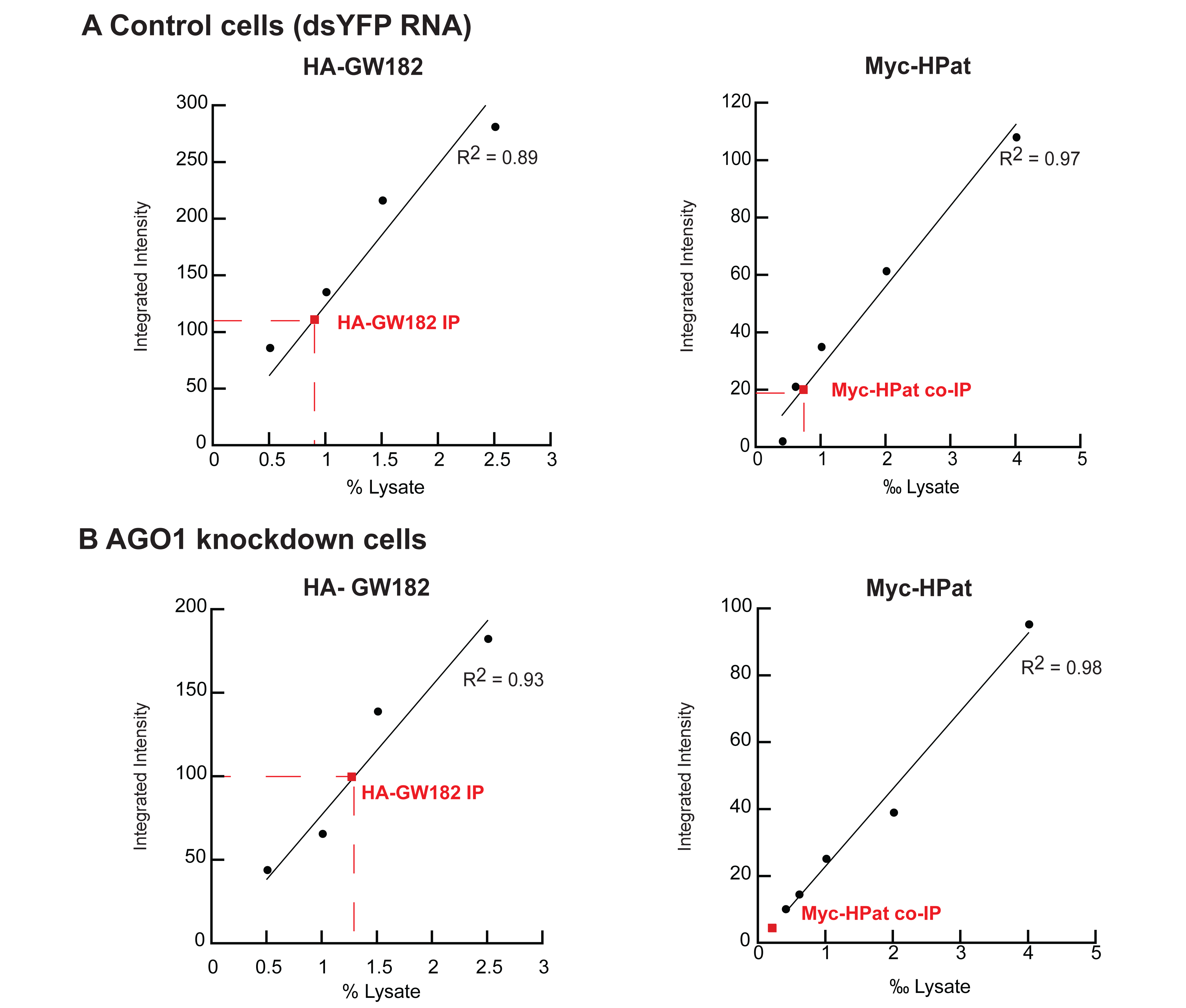

Supplement: Figure S2 — Quantitative analysis of the western blots shown in Figure 3A . Graphs for control cells treated with dsYFP RNA are shown in (A) and AGO1 knockdown cells in (B). The signal intensities were obtained using the Odyssey 2.1 (Li-Cor) and plotted against the amount of cell lysate. The amount of Myc-HPat or HA-GW182 in the immunoprecipitate was calculated relative to the amount of cell lysate in the input sample. (TIF) [file pone.0071860.s002.tif]

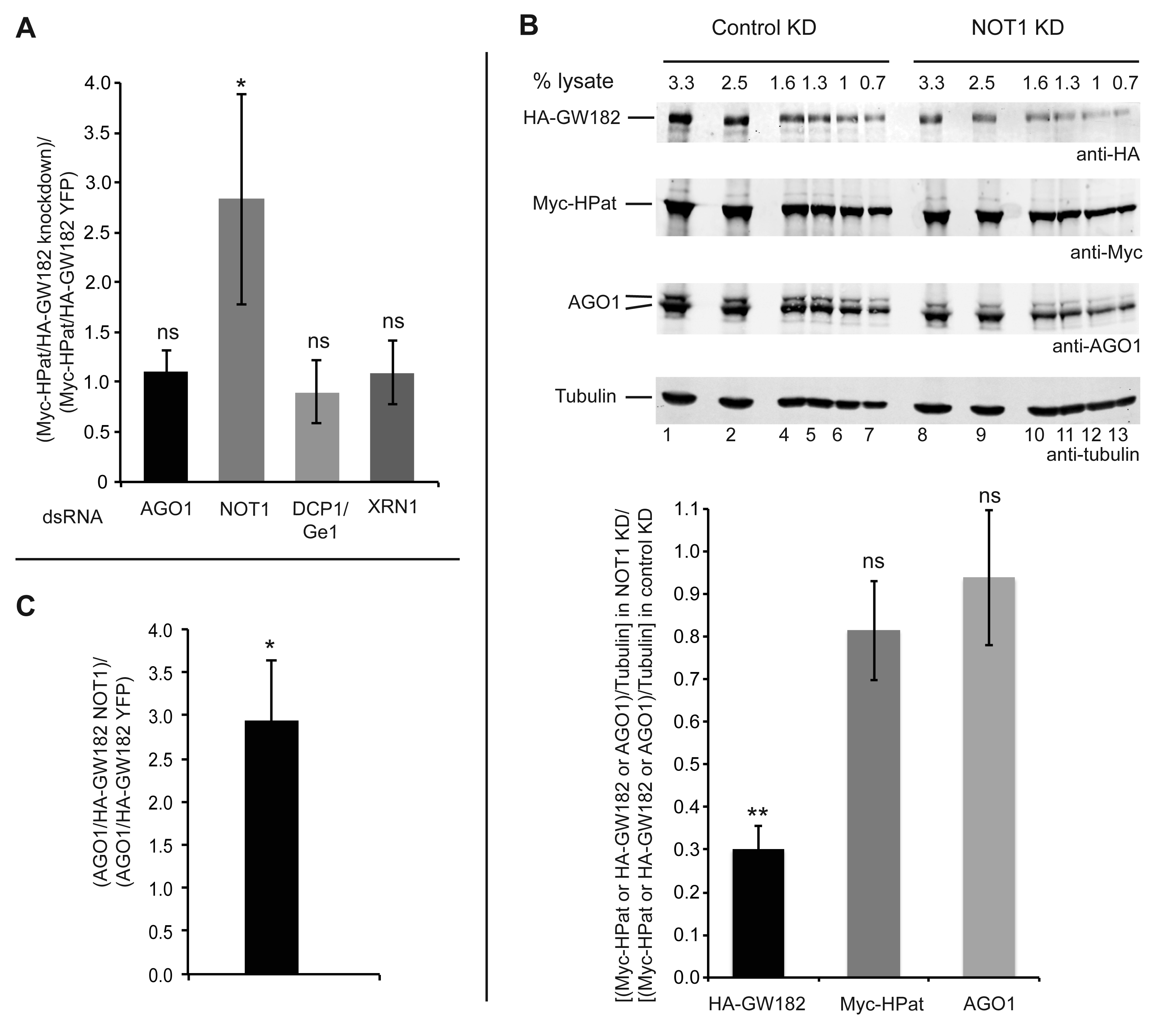

Supplement: Figure S3 — A, C: Relative expression levels of Myc-HPat/HA-GW182 (A) or AGO1/HA-GW182 (C) in different knockdown cells. The linear regression line of the quantitative input analysis of all biological replicates including Supporting Figures S2, S4 and S5 were used to calculate the ratio of Myc-HPat (A) or AGO1 (C) to HA-GW182 in input samples. As in all manuscript figures the bars represent the mean values of at least three independent biological replicates and the error bars the standard deviations. B: Protein levels of Myc-HPat, HA-GW182, and Tubulin in NOT1 and YFP knockdown cells. Increasing amounts of cell lysates from control cells (lanes 1–7) and NOT1 knockdown cells (lanes 8–13) were analyzed by western blot analysis using anti-HA, anti-myc, anti-AGO1 or anti-Tubulin antibody. The percentage of total cell lysate loaded is indicated. The graph below shows the quantitative analysis of the western blot. The signal intensities were obtained using the Odyssey 2.1 or ImageStudio (Li-Cor) and plotted relative to the amount of cell lysate. Values obtained from the linear regression were used to normalize HA-GW182, Myc-HPat, or AGO1 to Tubulin. These normalized values were used to calculate the ratio of HA-GW182, Myc-HPat, or AGO1 in NOT1 knockdown cells to control cells. For statistical analyses in A–C the Student’s t test was performed and the significances are as follows: ns, not significant; *, p<0.05; **, p<0.01. (TIF) [file pone.0071860.s003.tif]

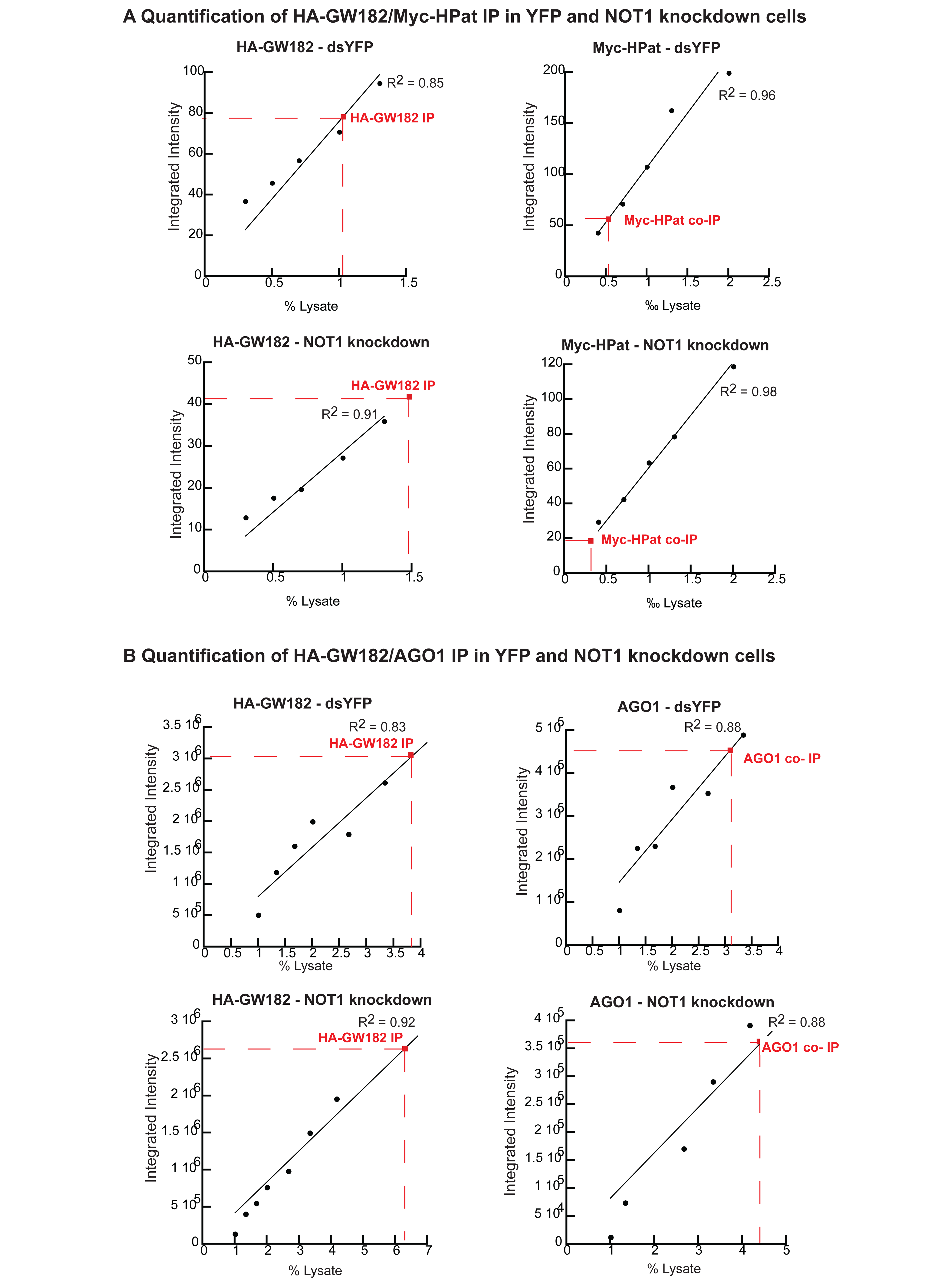

Supplement: Figure S4 — Quantitative analysis of the western blots shown in Figure 4A (A) and Figure 4B (B). The signal intensities were obtained using the Odyssey 2.1 (Li-Cor) and plotted relative to the amount of cell lysate. The amount of Myc-HPat or HA-GW182 in the immunoprecipitate was calculated relative to the amount of cell lysate in the input sample. (TIF) [file pone.0071860.s004.tif]

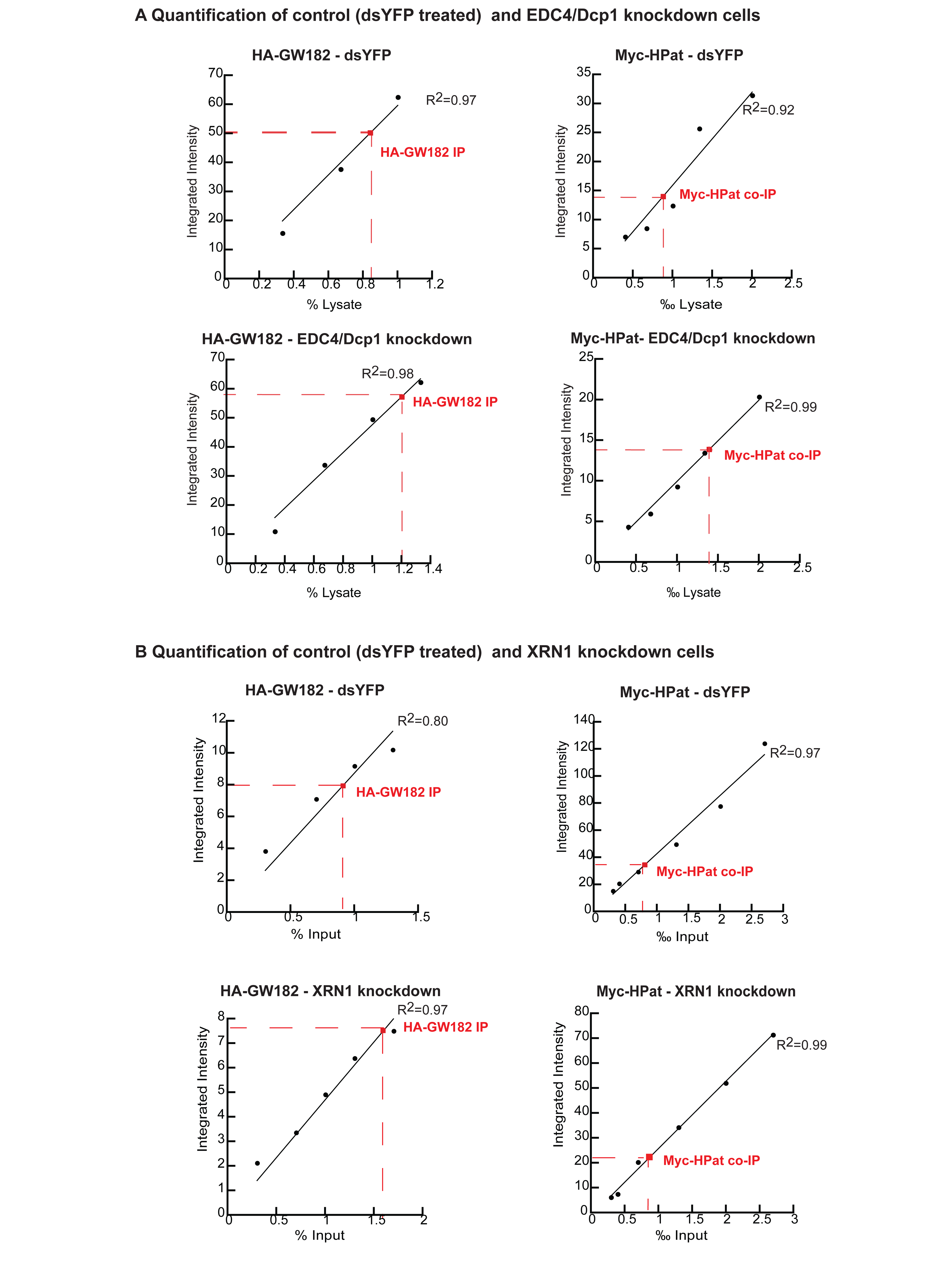

Supplement: Figure S5 — Quantitative analysis of the western blots shown in Figure 5A (A) and Figure 5B (B). The signal intensities were obtained using the Odyssey 2.1 (Li-Cor) and plotted relative to the amount of cell lysate. The amount of Myc-HPat or HA-GW182 in the immunoprecipitate was calculated relative to the amount of cell lysate in the input sample. (TIF) [file pone.0071860.s005.tif]
