# Supplementary material for: HPat a Decapping Activator Interacting with the miRNA Effector Complex
Source: PLoS One. 2013 Aug 19;8(8):e71860. doi: 10.1371/journal.pone.0071860 (PMC3747071; doi:10.1371/journal.pone.0071860)
Supplement: Table S1 — Primer sequences for PCR fragments for dsRNA synthesis. (PDF) [file pone.0071860.s006.pdf]

Primer sequences for PCR fragments for dsRNA synthesis

Target gene	Primer sequence 5' to 3'
DCP1 (SD301, fwd)	TAATACGACTCACTATAGGGAGATCGTGGATTCGTCCTCGCAC
DCP1 (SD302, rev)	TAATACGACTCACTATAGGGAGACGGAGATTGAGCCGTAGCGTAG
EDC4 (SD289, fwd)	TAATACGACTCACTATAGGGAGACGCTTGCACACTTGCCAATC
EDC4 (SD290, rev)	TAATACGACTCACTATAGGGAGAGCTGCTGCAGACATAGGGAC
NOT1 (SD196, fwd)	TAATACGACTCACTATAGGGAGAGCTCACTCAGCATCGCCATCG
NOT1 (SD197, rev)	TAATACGACTCACTATAGGGAGAGTAGGCGAAGGCCGACACAAT
XRN1 (SD299, fwd)	TAATACGACTCACTATAGGGAGATGAACTGATCGAGGAACTGTGCC
XRN1 (SD307, rev)	TAATACGACTCACTATAGGGAGACCAGCTGGCGCTTGCG
AGO1 (SD310, fwd)	TAATACGACTCACTATAGGGAGACATTAAAAAGCTGACCGATATGC
AGO1 (SD311, rev)	TAATACGACTCACTATAGGGAGA TTGACGTTGATCTTCAGACACAG
YFP (SD308, fwd)	TAATACGACTCACTATAGGGATGGTGAGCAAGGGCGAG
YFP (SD309, rev)	TAATACGACTCACTATAGGGAAGTTCACCTTGATGCC
