# Supplementary material for: HPat a Decapping Activator Interacting with the miRNA Effector Complex
Source: PLoS One. 2013 Aug 19;8(8):e71860. doi: 10.1371/journal.pone.0071860 (PMC3747071; doi:10.1371/journal.pone.0071860)
Supplement: Table S2 — Primer sequences for qPCR analysis. (PDF) [file pone.0071860.s007.pdf]

Primer sequences for qPCR analysis

Target gene	Primer Sequence 5' to 3'
EDC4 (fwd SD291)	CCAGGTGTGCAAGAGCAAGCAAT
EDC4 (rev SD292)	AAGCTGGAATCAAGGTGCTGCTCATA
DCP1 (fwd SD482)	CAGCCACTGCTGAACTCGAC
DCP1 (rev SD483)	GTAGGCCTTGTGCAGCTTGT
NOT1 (fwd SD466)	GTCCCTGAAGCCTGTTGCGGTA
NOT1 (rev SD467)	AGGAGTGGGTCAAGGCCAAGG
XRN1 (fwd SD592)	TTTCACCTGGAGGAGGAGCAGA
XRN1 (rev SD593)	CGCTGCGGCTTGATCAGGTA
Rp49 (fwd SD188)	CACAAATGGCGCAAGCCCAAGGGTATCG
Rp49 (rev SD189)	GGACCTCCAGCTCGCGCACGTT
CG6770 (fwd SD480)	GAGGCCCACTTCGATGAGTACG
CG6770 (rev SD481)	GTGCTCATTGGCCTCCCTCT
CG5123 (fwd SD575)	CAGCAGAGTGCCCCGCAAAT
CG5123 (rev SD576)	ATGGCCGTGCGGAAAGAACA
